# Barriers and facilitators of older adults for professional mental health help-seeking: a systematic review

**DOI:** 10.1186/s12877-023-04229-x

**Published:** 2023-08-25

**Authors:** Usra Elshaikh, Rayan Sheik, Raghad Khaled Mohammad Saeed, Tawanda Chivese, Diana Alsayed Hassan

**Affiliations:** 1 Department of Public Health, College of Health Sciences, QU Health, Qatar University, P.O. Box 2713, Doha, Qatar; 2 College of Medicine, QU Health, Qatar University, P.O. Box 2713, Doha, Qatar

**Keywords:** Mental health, Older adults, Help-seeking, Barriers, Depression, Anxiety

## Abstract

**Background:**

Older adults are at an increased risk for mental health issues, yet they are less likely to seek professional help. This systematic review aims to identify and summarize literature on the barriers and facilitators that older adults face when seeking professional mental health help.

**Methods:**

A comprehensive literature search was conducted using multiple databases including PubMed-Medline, EMBASE, ProQuest central, CINAHL and Scopus to identify relevant studies published between 2010 and 2021 that focused on barriers and/or facilitators to seeking help for depression, anxiety, and psychological distress among older adults aged 65 years or older. Studies’ risk of bias was assessed using the Newcastle–Ottawa Scale and results of studies were synthesized guided by the methodological framework of Rodgers and colleagues.

**Results:**

A total of eight cross-sectional studies, from Australia, United States, Mexico, Netherlands, and Malaysia met the inclusion criteria for this review. Included studies reported that the majority of their participants had anxiety or depression, yet they exhibited a preference for informal mental health help over professional help. Stigma, negative beliefs about mental health professional services, and cost were the most reported barriers. Main reported facilitators were prior positive experience with mental health services and high socioeconomic status.

**Conclusion:**

Older adults are in need of interventions normalizing mental health help seeking and ensuring these services are accessible in terms of costs. This should be the focus of policy makers, healthcare providers, and public health practitioners working with older adults.

**Protocol registration:**

PROSPERO 2021 CRD42021238853.

**Supplementary Information:**

The online version contains supplementary material available at 10.1186/s12877-023-04229-x.

## Introduction

It is estimated that 970 million people were living with a mental health disorder in 2019, which translates to more than one in eight people globally [1]. Contrary to the common believe, developing a mental health disorder is not a normal part of ageing [2]. Over 20% of those aged 60 and above suffer from a mental or neurological disorder, excluding headache disorders, and 6.6% of all disability adjusted life years (DALYs) among those aged 60 and older is attributable to mental and neurological disorders. These illnesses account for 17.4% of Years Lived with Disability among the elderly (YLDs) [2]. Despite the burden of mental health illness on older adults, they are unlikely to seek mental health help [3]. The reluctance to seek formal mental health treatment can lead to adverse outcomes that can only result in the worsening of the individual’s mental health status [4]. The lower the help-seeking, the higher the burden and costs of mental health issues and the lower the life expectancy [5].

According to the World Health Organization (WHO), the older adult population is estimated to double between 2015 and 2050 from 12 to 22% (WHO) [6]. Many of these older adults go through major life changes that can range from losing loved ones, retirement, and dealing with physical illnesses. Some older adults may adjust to these changes; however, others may find it challenging to adapt making them more vulnerable to the development of mental health conditions such as depression and anxiety.

Given the serious burden of mental health illness on older adults and the fact that they are an under-researched group due to their limited use of mental health services [7], further studies are needed in order to add to the literature on the barriers faced by older adults deterring them from seeking professional help.

Mental health help seeking is communicating the need for personal and psychological assistance to obtain advice and support from formal care [8]. Furthermore, seeking mental health help is an adaptive process, in which the person attempts to obtain external assistance to deal with mental health concerns in order to have the ability to cope and adjust with new experiences with the least conflicts [9]. In our study, seeking help was defined as actively engaging with at least one professional service, such as reaching out to a mental health hotline, consulting a healthcare professional, or using online mental health services provided by professionals. In our study, barriers toward mental health help seeking was defined as factors that cause a delay or avoidance to seeking formal mental health care. On the contrary, facilitators were identified as factors that not only motivate, but also make it easier for older adults to seek mental health help. Therefore, the aim of this study is to systematically review and summarize the literature on the barriers and facilitators that influence older adults’ mental health help seeking for anxiety, depression, and general psychological distress; the most prevalent mental health conditions among this vulnerable group.

## Methods

We conducted this systematic review in accordance with the Preferred Reporting Items for Systematic Reviews (PRISMA) guidelines [10]- available at (Additional file 1). We designed a review protocol, developed a data extraction form, and then registered at the International Prospective Register of Systematic Reviews (CRD42021238853).

### Search strategy

We conducted a systematic search using four research databases with no language or place restrictions. The databases included were PubMed-Medline, EMBASE, ProQuest central, CINAHL and Scopus. In addition, Google Scholar, article references, and relevant reviews were further searched and investigated to achieve a comprehensive search for any additional relevant publications. Our search strategy was based on synonyms of the primary search terms, which included four main concepts: mental health, AND help-seeking, AND barriers, AND/OR facilitators. These terms were used with a comprehensive list of key words and Medical Subject Headings (MeSH) terms to identify potentially relevant articles, available at (Additional file 2). The search was limited to studies from 2010–2021 and conducted on March 10, 2021.

### Screening of studies

One author exported the articles to endnote and removed duplicates. At least two authors performed an independent assessment for eligibility using Rayyan; a web-tool for screening of abstracts and titles. Disagreements between the reviewers were resolved by a discussion under the supervision of a third author. Full article screening was conducted, again independently by two authors per publication, for eligibility.

### Eligibility criteria

We included studies that met the following inclusion criteria: 1) published during the period between 2010 and March 2021, 2) studies that addressed barriers and/or facilitators. This includes studies that examined correlations between barriers/facilitators and mental health help-seeking among older adults and/or studies that quantified the proportion of older adults reporting specific barriers or facilitators 3) examined depression, anxiety, and psychological distress disorders and 4) quantitative, population-based, and observational study designs. We excluded studies that met the following criteria: 1) exclusively qualitative studies, systematic reviews, experimental studies, and 2) examined organic mental health disorders such as dementia, Alzheimer’s, schizophrenia, acute organic brain syndrome, delirium, amnestic syndrome, personality disorders, or any mental health disorders due to physical brain damage or physiological dysfunction.

### Data extraction and assessment of study quality

We piloted and used a standardized form to extract the following information for each included study. We extracted the following data for each study: study title, study author/s, year, design, sampling approach, age, sample size, aim of the study, setting, region/country, gender, population description, definition of mental health help-seeking, type of mental health condition assessed, possible conflict of interests, publication type, barriers, facilitators, any effect estimates and their sizes, confounders adjusted for, 95% CI for effect estimates, and the percentages for people willing to and/or currently seeking help. Two authors extracted data for each study, then compared their data. We assessed and scored the risk of bias of the included studies using an adapted version for cross sectional studies, the Newcastle–Ottawa Scale (NOS) [11]. Two reviewers independently appraised the quality of the included studies, grading the studies out of 10 points. Afterwards we resolved any existing conflicts by meeting to reach an agreement. We used Newcastle–Ottawa Scale to assess the internal and external validity of studies by evaluating appropriateness of selection and recruitment of subjects, representativeness and comparability of subjects based on study design or analysis, response rate, methods for ascertainment of exposure and outcome, and appropriateness of statistical analysis. Four cut-off points were considered to assess the quality of the studies: A cross-sectional study with a score of 9–10 was considered as high quality with minimum risk of bias, 7–8 was considered good quality with a moderate risk of bias, 5–6 was considered satisfactory with a high risk of bias, and 0–4 was considered unsatisfactory study with a very high risk of bias [11]. The NOS used is provided in (Additional file 3).

### Synthesis of results

Given the nature of the research question and the variety of measures employed in assessing barriers and facilitators across studies, conducting a meta-analysis was deemed unfeasible. Instead, the authors elected to conduct a narrative synthesis to analyze the findings. To eliminate bias and promote openness, the authors followed the methodological framework of Rodgers and colleagues that provides guidance on conducting rigorous narrative synthesis when statistical meta-analysis is not feasible or advised [12].

To identify the most important themes, a rigorous and systematic approach was employed. The included studies underwent a comprehensive review, wherein the findings were carefully analyzed after data extraction to identify recurring themes. Two criteria were considered during the evaluation process to determine the significance of these themes. Firstly, the frequency of mention of specific barriers and/or facilitators across the studies was taken into account. Themes that were consistently reported by a substantial number of studies were deemed to be of high importance. Additionally, the percentage of the population within each study that reported these barriers or facilitators was considered. Themes that were consistently reported by a larger proportion of the population across studies were given greater attention and regarded as significant factors influencing help-seeking behaviors. By incorporating these criteria, we aimed to identify the most salient and impactful barriers and/or facilitators that emerged from the included studies.

## Results

### Study selection

**Fig. 1:**
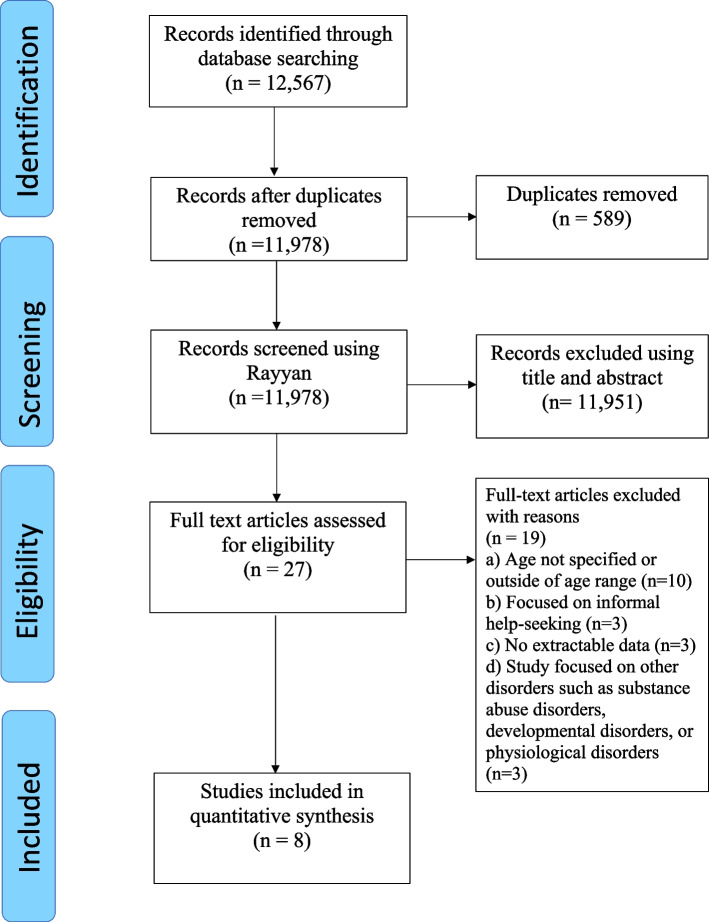
PRISMA flow diagram

As we illustrated in Fig. 1, we imported 12,567 study records yielded from the search to Endnote, removed 589 duplicates, selected 27 study records for full text screening, and finally excluded 19 studies that did not meet the inclusion criteria, leaving a total of 8 studies eligible for inclusion. The reasons for the exclusion of the 19 studies were a lack of data on age (*n* = 10), informal help seeking (*n* = 3), data not being extractable (*n* = 3), focused on other type of mental disorders (*n* = 3).

### Study characteristics

The characteristics of the studies included in this systematic review are presented in Table 1. Four of these studies were conducted in the U.S.A [7, 13–15], one in Malaysia [16], one in the Netherlands [17], another in Mexico [18], and one in Australia [19]. All eight studies were cross sectional in design. Three studies addressed community dwelling older adults [14, 18, 19] with one in residential care units [19] and the other in a rural community [14]. Sample sizes ranged between 105 [19] to 93,938 participants [7]. Three studies examined both anxiety and depression [7, 13, 14], one study examined anxiety only [19], another examined psychological distress [15], and three studies examined depression only [16–18].

**Table 1:** Characteristics of studies included in the systematic review

Author, year	Country	Study design	Population and setting	Sample size	Gender %	Age (mean, SD) Or Age range	Mental health disorder	Percentage of people who have sought, or are actively seeking, or willing to seek mental health help	Main findings
Anderson et al., 2017 [19]	Australia	Cross sectional. (Survey)	community-dwelling older people in residential care units	105	69.2% females 30.8% males	(79.4, 7.1)	Anxiety	81.80% reported that they would seek help if they suspected they were experiencing anxiety 60% would request help from their primary care physician	About half of the participants (47.00%) reported concerns about various aspects of seeking help for anxiety Worries about the cost, privacy, diagnosis, anticipating negative perceptions and judgment from others (even health professionals), doubts about GPs' capacity to manage anxiety, concerns about medication consumption, and lack of knowledge with the process were among them
Chai et al., 2021[16]	Malaysia	Cross sectional- in person survey	Elderly patients at a primary care clinic	273	Male 47.6% Female 52.6%	(69.9, 6.9)	Depression	8.8% (*n* = 24) sought help from professionals 32.1% (*n* = 9) of respondents who were diagnosed as depressed using the PHQ-9 (n = 28) had plans to seek professional care for their depression	With an adjusted odds ratio (OR) of 3.45 and a 95% confidence interval (CI) of 1.41 to 8.48, prior experience seeking professional help was related with an increased likelihood of requesting treatment Respondents with secondary education had an adjusted OR of 3.10 and a 95% CI of 1.01 to 9.53, while those with tertiary education had an adjusted OR of 4.66 and a 95% CI of 1.08 to 20.04, compared to those with no formal education
Brenes et al., 2015 [14]	USA	Cross sectional- (telephone screening)	Older adults living in rural communities	478	Women 77.4% Men 22.6%	(68.4, 7.1)	Anxiety / depression	75.3% (n = 360) reported that they needed help with anxiety or depression in the last year	80.1% of respondents believed they "should not need help," whereas 41.9% distrusted mental health experts and 41.2% did not want to discuss personal concerns with a stranger Furthermore, 40.0% did not believe that treatment would be beneficial. Cost (58.4%) and not knowing where to go (49.6%) were also mentioned as practical constraints Embarrassment (39.8%) and anxiety about others' opinions (39.8%) were among the stigma-related barriers The results revealed a negative relationship between age and the number of reported barriers, with a coefficient (standard error) of -0.06 (0.02), t-value of -2.96, and p-value less than 0.01. Worry severity, on the other hand, was connected with the number of reported barriers, with a coefficient (standard error) of 0.09 (0.02), t-value of 3.98, and p-value less than 0.0001
Blais et al., 2015 [13]	USA	Cross sectional- Secondary data analysis from a previously conducted study (National Health and Resilience in Veterans Study)	Older U.S. veterans	2,025	Male 97% Female 3%	(71, 7.1)	Depression, anxiety, PTSD	16% (*n* = 332) Of the full sample of veterans (distressed and un distressed) reported that they have utilized mental health treatment 6% (*n* = 130) Of the full sample of veterans reported that they are currently utilizing mental health treatment	In a multivariate model, the use of mental health treatments among distressed veterans was found to be negatively linked with increasing age Nevertheless, only current suicidal ideation remained independently linked with usage in the same model. Also, in multivariable analyses, unfavorable perceptions about mental health care were negatively connected with service utilization among distressed veterans
Sorkin et al., 2016 [7]	USA	Cross sectional- secondary data from 2007, 2009, 2011–12, and 2013–14 California Health Interview Survey (CHIS)	Older adults	93,938	Female 54.8% Male 45.1%	Range of means: (64.6 – 67.4)	anxiety, severe cognitive impairment, depression	NR	When compared to non-Hispanic Whites (NHWs), Asian and Pacific Islanders (APIs) and Blacks were much more likely to report worries about someone finding out as a reason for not seeking treatment Furthermore, API and Hispanic respondents were more likely than NHW respondents to report concern with speaking with a professional as a reason for avoiding seeking therapy
Holvast et al., 2012 [17]	Netherlands	Cross sectional- secondary data from the Netherlands Study of Depression in Older persons (NESDO) and the Netherlands Study of Depression and Anxiety (NESDA)	Older persons/primary care practices	167	Males: 35.3% Females: 64.7%	Weighted mean: 61.9 Range: (55–87)	Depression	70% (*n* = 117) of respondents reported having contact with an individual regarding psychological concerns in the preceding 6 months	People who were born in the Netherlands, and individuals who felt less lonely, and those who had a greater household income had a higher chance of receiving mental health care as they aged
Sorkin et al., 2011 [15]	USA	Cross sectional- data collected from the California Health Interview Survey	population- based sample of Asian American older adults	1,606	Males: (44.3%) Females: (55.7%)	Range of means: (64.6–71.3)	Psychological distress	Korean (1.6%), Japanese (1.0%), and Chinese (1.0%) older adults also were significantly less likely to see their primary care physician compared to non-Hispanic whites (6.5%). Similar results for other mental health professionals	Filipino Americans and Korean Americans had more than twice the likelihood of experiencing symptoms of mental distress as compared to non-Hispanic Whites Yet, they were less likely to have taken prescription medication or seen a primary care practitioner In comparison to non-Hispanic Whites, Japanese Americans were less likely to report symptoms of mental distress and less likely to use mental health services
Pérez-Zepeda et at., 2013 [18]	Mexico	Cross sectional	community dwelling elderly belonging to the largest health and social security system in Mexico	2,322	Males: 32.86% Females: 67.14%	(73.18, 7.02)	Depression	57.92% (*n* = 1345) reported depressive symptoms in the last year Out of those who reported depressive symptoms only 25% (n = 337) did seek help	Women and people who believe depression is not a disease are less likely to seek mental health treatment Education level and recent usage of the healthcare system, on the other hand, were shown to be characteristics that assisted the receipt of specialized mental health care

### Risk of bias of included studies

Reviewers rated three of the studies included as satisfactory quality, two were classified as good, and three studies as very good. However, all of the studies were observational, which provides very limited evidence for the association between the given factors and professional help-seeking. provided the independent component scores and overall ratings for all studies in (Additional file 4).

### Help-seeking of mental health services

As shown in Table 1, out of the eight included studies, seven studies addressed participant’s willingness, need and current use of mental health services [7, 14, 16–19]. One study from Australia reported that 81.8% (*n* = 86) of the community-dwelling older adults in their sample would seek help if they perceived that they were experiencing anxiety [19]. The second study, conducted in Malaysia, reported that 76.9% (*n* = 210) of the elderly patients in their study sought help from others regarding their emotional problems [16]. This percentage included informal mental health help resources such as friends, family, and religious organizations. On the contrary, seeking professional help from counsellors, social workers, psychologists, and health practitioners accounted for only 8.8% (*n* = 24) of the total sample population [16]. The third study was conducted by Brenes et al. (2015) in the U.S.A among rural older adults and found that 75.3% (*n* = 478) of the participants needed help in the past year with anxiety or depression [14]. Despite this high percentage, when participants were screened for the barriers, 80.1% (*n* = 358) endorsed the belief of “I should not need help” [14]. The fourth study conducted by Holvast et al. (2012) in the Netherlands found that 70.1% (*n* = 117) of the respondents had contacted professionals in regards to psychological problems in the last 6 months [17]. Pérez-Zepeda et al. (2013) also conducted a study and found that only 25.06% (*n* = 337) sought help [18]. Blais et al. (2015) conducted a study in the US, which found that out of the full sample that included old veterans, only 16% (*n* = 332) reported that they utilized mental health treatment in their lifetime, and 6% (*n* = 130) reported that they are currently utilizing mental health treatment [13]. Sorkin et al. (2011) conducted a study in the USA and found that Filipino Americans (aOR = 0.41; 95% CI = 0.18–0.9), Korean Americans (aORs range = 0.15 to 0.24) and Japanese Americans (aORs range = 0.16 to 0.18) were less likely to make use of any mental health services, including seeing a primary care provider/other professional compared to non-Hispanic whites [15].

### Reported facilitators and barriers

#### Stigma

Three out of the eight studies included found that stigma was one of the main barriers that hinders the process of mental health help-seeking. Brenes et al. (2015) from the USA, published a study with a total of 478 participants and 77.4% females and found that 39.8% of their participants reported embarrassment and worry about what others would think as a barrier [14]. Blais et al. (2015) from the USA, published a study with 2,025 participants, 97% male, and found in a multivariable model, perception of stigma showed a negative association with utilization of mental health care (OR = 0.80, 95% CI = 0.68–0.93) [13]. After a post hoc analysis, it was found that the association was driven by the statement “I would be seen as weak” (OR = 0.25, 95% CI = 0.11–0.58) [13]. Anderson et al. (2017) from Australia, published a study with a total of 105 participants and 30.8% males, found that the overall level of stigma was low. The only exception was that 53% of participants felt that individuals who are anxious make them feel uncomfortable [19].

#### Age

Age was reported in three studies. Blais et al., reported that older age was found to have a negative association with utilization of mental health among distressed veterans (OR = 0.89, 95% CI = 0.81–0.97) [13]. However, another study from the Netherlands, published in 2012, with a total of 167 participants and 64.7% males reported that the odds of having medical contact for mental health problems increased with advancing age (OR = 1.07, 95% CI = 1.00–1.15) [17]. A different study found that age was negatively associated with the number of barriers reported (Coefficient [Standard Error] =  − 0.06 [0.02]; t =  − 2.96, df = 452, *p* < 0.01) [14].

#### Cost

A total of two studies found cost as a barrier. A study from Australia, published in 2017, with a total of 105 participants and 30.8% males, stated that 47% of their participants reported at least 1 barrier including cost among other different barriers barrier to help-seeking for anxiety [19]. Cost has also been reported in another study, in which more than half of their participants (58.4%) endorsed cost as a barrier [14]. On the other hand, a study examining barriers to mental health help-seeking among a diverse large sample from the USA, published in 2016, with a total of 93,938 participants, 54.8% females, found that there were no ethnic or racial differences in cost being cited as a reason for not seeking treatment (F(3,320) = 0.26, *p* = 0.85) [7].

#### Race/ethnicity

Out of the included studies, only two assessed race as a factor for professional mental health help-seeking. A study conducted in the US, during 2011, with a total of 1,606 participants and 55.7% females stated that Filipino- and Korean Americans were more likely to report symptoms of mental distress compared to non-Hispanic whites (aOR = 2.25, 95% CI = 1.14–4.47) and (aOR = 2.10, 95% CI = 1.06–4.17), respectively [15]. However, they were less likely to have seen a medical healthcare provider [Filipino: aOR = 0.41; 95% CI = 0.18–0.90; Korean: aOR = 0.24; 95% CI = 0.08–0.69] or have taken a prescription medication [Filipino: aOR = 0.20; 95% CI = 0.10–0.40; Korean: aOR = 0.15; 95% CI = 0.05–0.40].

In a different study conducted in the US as well, published in 2016, with a total of 93,938 participants, 54.8% females highlighted racial and ethnic differences in reasons for not seeking care, racial differences were significant in the odds of endorsing whether respondents felt comfortable talking with a professional about their personal problems (F(3,320) = 2.91, *P* = 0.03) [7]. Additionally, there were significant ethnic and racial differences in the odds of endorsing feeling concerned about someone finding out about a mental health issue (F(3,320) = 8.99, *P* < 0.001). Hispanic respondents have more than twice the odds of reporting having a hard time getting an appointment compared with non-Hispanic whites (NHW) respondents (aOR = 2.3, 95% CI = 1.1–4.7, *P* = 0.02) (Sorkin et al. 2016). Hispanic respondents had lower odds of endorsing concerns about someone finding out compared with Asian and Pacific Islander (API) respondents (aOR = 0.2, 95% CI = 0.1–0.6, *P* < 0.001). Both API (aOR = 5.5, 95% CI = 2.2–16.3, *P* < 0.001) and black (aOR = 3.5, 95% CI = 1.6–7.6, *P* = 0.002) respondents had significantly higher odds of endorsing concerns about someone finding out as a reason for not seeking treatment compared with NHW respondents. API and Hispanic respondents were more likely than NHW respondents to report concerns about not feeling comfortable talking to a professional as a reason for not seeking treatment [7]. A different study conducted in the Netherlands, reported that participants born in the Netherlands had higher odds of having contact with the healthcare system for mental health problems compared to not being Dutch-born (OR = 3.17; 95% CI: 1.18– 8.52) [17].

#### Gender

Gender was found to have an independent association in one study [18]. The study was conducted in Mexico, published in 2013, with a total of 2,322 participants, 67.14% females [18]. The study found that males exhibited a positive help-seeking behavior more than females [18]. However, this association was only observed significantly in the stepwise model that authors explain as a possible type 1 error (female gender OR = 0.07 95% CI 0.511–0.958 *p* = 0.026) [18].

#### Education

Two studies found that level of education is a facilitator for professional mental health help-seeking. One study was conducted in Mexico, published in 2013, with a total of 2,322 participants, 67.14% females reported that education is associated with receiving specialized mental health care for depression among community dwelling older adults (OR = 1.065, CI 95% 1.04–1.104) [18]. Another study among a sample of elderly in Malaysia published in 2021, with 273 participants, and 47.6% males found that individuals with a secondary and tertiary education had the highest odds to seeking professional help for depression compared to individuals with no formal education (aOR = 4.66, 95% CI = 1.08–20.04) [16].

#### Prior experience of seeking professional help

Prior experience of seeking professional help was reported in one study out of the eight included studies [16]. The study was conducted in Malaysia, published in 2021, with 273 participants, and 47.6% males. The study found that prior experience of seeking professional help was associated with seeking professional help for depression (aOR = 3.45, 95% CI = 1.41–8.48) [16]. A detailed summary of the main findings of each study can be found in Table 1.

## Discussion

This review examined factors that can either assist or hinder older adults in seeking mental health professional help. The most prominent factors reported throughout the studies were stigma, negative beliefs of the effectiveness of mental health treatment, cost, socioeconomic status, age, and prior experiences related to mental health help-seeking.

Multiple studies found that the majority of participants expressed concerns about the stigma surrounding mental health [13, 14, 19]. Despite not being a standalone barrier in some studies, participants cited reasons for not seeking help such as worrying about other people's opinions and being concerned about someone finding out, indicating the influence of stigma. For example, in some studies participants endorsed reasons for not seeking help such as “I was worried about people’s opinion about me” [18], “Concerned about someone finding out” [7]. Mental health stigma can be defined as profound negative stereotypes about people living with mental disorders [20].

Stigma can be divided into internal or external, in which external stigma refers to the public’s unfavorable views, attitudes, and perceptions about mental illness, that contribute to stereotyping, prejudice, and discrimination towards people with mental health conditions [21]. While internalized stigma, also known as self-stigma, is defined by a subjective sense of devaluation, marginalization, secrecy, shame, and withdrawal by applying negative stereotypes to oneself [22]. People in general feel uncomfortable being around people with anxiety [19]. Thus, Stigma and generational taboos can inhibit the process of mental health help-seeking by taking a part in alienating those who need mental health services. A systematic review and meta-analysis found that individuals with personal stigma towards others with mental illness were less likely to engage in active help-seeking [5]. The type of stigma determines the intensity of the association, as it was found that participant’s level of internal stigma and attitudes towards help-seeking were associated with less active help-seeking for mental health [5]. A study conducted with a national sample of Canadian participants aimed to examine the variables associated with mental health help-seeking revealed that public stigma of seeking help did not have a significant impact on help-seeking behavior [23]. However, it was observed that self-stigma of seeking help exerted a strong negative influence, indicating that individuals' own internalized stigma played a critical role in their decision to seek help for mental health issues [23]. Individual's negative impressions of society's attitudes and ideas about mental health lead to negative attitudes towards treatment, creating a barrier to seeking help [5]. People who hold stigmatizing views towards others with mental illness, tend to avoid contact with that group and refrain from seeking help. This is in line with the findings from Blais et al. (2015), where those who had high perception of stigma tended to have low levels of mental health services utilization [13]. Similarly, in Brenes et al. (2015) participants showed an extremely high level of reluctance to seek mental health help, due to internalized stigma [14].

As reported in literature, stigma associated with mental health can impact help-seeking among people of different races and ethnicities. A 2014 systematic review found that ethnic minorities, such as Asians, Arabs, and African Americans, are more likely to face stigma and, as a result, seek less help [24]. A different 2020 systematic review also reported that racial minorities, such as Black (African background), Asian, Hispanic (Latin American and Spanish background), and Native American (referring to North American indigenous people) have higher mental illness stigma than racial majority [25]. This can be explained by the collectivist nature of these societies and the belief that mental health problems can be solved without professional treatment. These reviews highlight the need for culturally sensitive and responsive mental health treatments that address the unique barriers to help-seeking faced by minority groups.

Stereotypes are not limited to fear and shame of seeking help, but it may also affect how people perceive the effectiveness of mental health treatment. Negative beliefs about mental health care can be explained by having the assumption that seeking professional help would be equal to or even worse than not seeking any help at all. Attitudes and beliefs about mental illness are shaped by personal knowledge, engaging with or knowing someone living with mental illness, culture, stereotypes, societal assumptions, media, and many other factors [26]. In accordance with our findings, negative beliefs about mental health care are affecting older adults’ willingness to seek mental health services [13, 14]. According to (Blais et al., 2015) negative beliefs about mental health care are associated with a decrease in mental health services utilization among distressed veterans [13]. While veterans may face unique barriers. Similar findings were reported in another systematic review that highlighted the effect of negative beliefs on predisposing factors on adult trauma survivors [27]. Kantor et al. (2017) reported that in some studies, adult trauma survivors were convinced that treatment is not effective, which deterred them from seeking mental health treatment [27].

Cost of mental health services, including treatments and out of pocket costs, is another burden faced by some older adults [14, 28]. Brenes et al. (2015) study was conducted in a rural population [14]. Cost of mental healthcare services is highly influenced by geographical factors and resource accessibility. Ziller et al. (2010) found that rural patients had poorer access to mental health treatments compared to urban patients despite having equal socioeconomic status, insurance status, and demographic features [29]. According to Ziller et al., this was attributed to the 'well-documented and persistent issues of mental health practitioner availability' for rural communities. Patients and clinicians in rural areas have a significantly greater cost burden. It has been reported that there is a disparity in insurance policy coverage between rural and urban areas, resulting in increased poverty in rural areas and inadequate coping mechanisms among rural populations. Ziller et al.(2010) found that rural residents spend more money on mental health than urban residents [29]. This could be because insurance coverage in rural regions is less comprehensive. Cost was also reported as a structural barrier to mental healthcare among other different populations in other systematic reviews [8, 27, 30, 31]. However, Lavingia (2020) found that intrinsic barriers, such as a lack of perceived need for treatment, have a larger influence than extrinsic barriers including cost in preventing patients from making the initial steps toward seeking healthcare [31].

Based on our findings, age can both be a barrier and a facilitator to seeking help for mental health issues. One study found that older age is associated with less barriers to seeking mental health services [14]. Elderly people may perceive less hurdles to mental health care as a result of skills and strategies acquired over the years to overcome barriers to seeking the care they need. As people age and have more health concerns, they have fewer competing demands for their time and resources. Similarly, another study found that odds of having contact for mental health problems increased with age [17]. Moreover, the predominance of the youngest age group (55–64) in that study may have contributed to this encouraging finding, as aging in this group of younger elderly is linked with a reduction in tasks due to retirement and suffering physical deterioration [17]. In a study conducted by Mackenzie & Pankratz (2022) among a large national sample of older adults, it was found that mental health service utilization decreased with age [23]. Specifically, the study revealed that only 23% of adults aged 75 and older reported using mental health services in the past five years, whereas 46% of adults between the ages of 55–64 reported the same [23]. These findings indicate a significant decline in mental health service utilization among older adults as they advance in age. Further explaining these findings, a study conducted to compare age difference and their help-seeking attitudes found that the attitudes of older adults toward obtaining professional help were generally more positive than those of younger adults, contributing to a small but rising body of literature refuting the ageist premise that older people avoid seeking professional help due to stigma and negative attitudes [32]. In terms of the positive impact of age on help-seeking tendency, older adults may be more likely to endorse statements like "I would want to get professional help if I were worried or upset for a long period of time" and "It would be relatively easy for me to find the time to see a professional for psychological problems" as they are less likely than younger individuals to idealize societal norms emphasizing strength and self-reliance, or because life experiences may have made them more open to seeking help [32].

In contrast to the findings of the previous studies, Blais et al., (2015) indicated that old veterans' usage of mental health services decreases with age, which can be explained by their negative views regarding mental health, particularly their fear of being viewed as weak or a burden on others [13].

Social inequalities in mental health care utilization continue to be highlighted as barriers to mental healthcare services utilization. Older adults with lower socioeconomic status may have less access to healthcare services due to financial constrains or lack of insurance [17]. They might also face additional stressors related to poverty, which can exacerbate mental health issue affecting their help-seeking behaviors.

Stigma has also been found to be more prominent in older adults with lower education levels [18]. Furthermore, high education level is positively correlated with mental health literacy [33]. Level of education can also play a role in older adults’ help-seeking for mental issues where the higher level of education is a facilitator [16, 18]. Accordingly, high education level reduces perceived self-stigma and gives a space for people to seek mental health help [30, 33] while people with a lower education level might be more likely to hold negative beliefs regarding mental illness thus hindering them from seeking professional help [34, 35].

Past experiences of obtaining mental health services is a key element in the process of seeking professional mental health services, as it can either promote the recurring desire to seek mental health help in the future or hinder it [16]. A positive experience may enhance beliefs in the success of treatment effectiveness and may improve the intention to seek help [16]. This suggests that people who have a history of mental health referrals may use active help-seeking strategies when they need it. Similarly, a study found that having experience in the mental health field, whether through volunteer work, peer help, or taking mental health courses, was associated with greater psychological openness and help-seeking willingness [36].

### Implications for practice and research

The processes by which older people seek treatment for mental health concerns are complex. Individual characteristics and attitudes, the frequency and severity of symptoms, contextual factors, and societal factors all contribute to the help seeking process. Findings from multiple studies, including ours, found that internal barriers, such as the lack of perceived need for treatment and stigma, had a greater influence on the initial steps toward seeking mental health help among older adults than extrinsic barriers, such as cost and accessibility [31]. Thus, focusing on providing interventions that target these internal barriers is crucial in improving the outcomes of mental health help seeking behaviors among older adults. A review on effective interventions to reduce mental health stigma found that social contact, defined as contact between people with and without mental illness, produce the most profound impact on mental health stigma; however, the impact has been shown to be effective on the short-term only [37]. A number of studies found that health professionals training in health advocacy, anti-stigma competency, or related skills is effective in tackling stigma [38, 39]. The integration of knowledge and contact-based approaches has proven to be a powerful combination in reducing stigma and increasing mental health knowledge [40, 41]. This can be seen in hearing first-person testimonies from people who have lived with a mental illness and have been trained to speak about their illness and recovery, as well as their experiences within the healthcare system. This is a key strategy for interprofessional educational approaches to stigma reduction in healthcare [40, 41]. In social contact approaches, people who have lived with a mental illness are considered as educators rather than patients that have been able to dispel misconceptions, reduce anxiety, increase empathy, foster personal relationships, and enhance knowledge of recovery [40, 42]. Individuals with mild to moderate mental health problems prefer informal sources of help in addition to general advice from their general practitioner since they are usually the first and safest option older adults may resort to when seeking formal mental health help [43]. Thus, investing in improving the quality of services and education given to them may be a cost-effective and beneficial approach to increase formal help.

Future research should focus on conducting more studies to understand the help-seeking behaviors of older adults. There is a need to develop and evaluate interventions that specifically address internal barriers to help-seeking, such as stigma, as it is one of the most frequent barriers among older adults. Additionally, comparative studies across diverse cultural and socioeconomic contexts would provide insights into variations in help-seeking behaviors and inform the development of culturally sensitive strategies. Furthermore, exploring the reasons behind older adults' preference for informal sources of help and improving the quality and accessibility of professional mental health services should also be a focus. Incorporating these recommendations would deepen our understanding of older adults' help-seeking behaviors and guide the development of targeted interventions and policies to enhance mental health support for this population.

### Strengths and limitations

This systematic review utilized a comprehensive search strategy and the most recent compilation of relevant up-to-date data from 2010 to March 2021. Where a sufficiently broad terminology list has been used, in addition to hand-searching of reference lists located some further papers not captured in the database searches Also, this systematic search has a robust design guided by the Preferred Reporting Items for Systematic Reviews (PRISMA) guidelines. This review has several limitations that need to be taken into consideration. In addition, some studies had an age range starting with a number less than 65, which is our defined population’s age. Furthermore, the heterogeneous measurements between the studies; there was a variation between the included studies’ outcome’s effect measures due to the variability found between healthcare systems, cultures, geographic locations, and contexts that shape perceptions. This limits the generalizability of our findings. The barriers and facilitators considered in this systematic review were reported by older adults, which might mean not being aware of all the potentially influential factors.

## Conclusion

In conclusion, this systematic review has identified a number of barriers and facilitators that older adults encounter while seeking professional mental health services. Stigma, high costs, and negative perceptions of mental health services have been recognized as major barriers. On the other hand, socioeconomic status and past positive experience concerning mental health have emerged as facilitators. The findings of this review suggest that interventions aiming at addressing these barriers and facilitators may be beneficial in enhancing the help-seeking behavior of older adults. Future research should focus on developing and analyzing such strategies, as well as investigating the unique needs of older adults seeking mental health care.

### Supplementary Information

**Additional file 1.** Preferred Reporting Items for Systematic Reviews and Meta-Analyses Checklist.

**Additional file 2.** Detailed search strategy used to identify relevant studies.

**Additional file 3.** NEWCASTLE - OTTAWA QUALITY ASSESSMENT SCALE.

**Additional file 4.** Newcastle-Ottawa Scale of included cross-sectional studies.

## Data Availability

The data supporting the findings of this study are available within the article and its additional files.
