# Supplementary material for: Barriers and facilitators of older adults for professional mental health help-seeking: a systematic review
Source: BMC Geriatr. 2023 Aug 25;23:516. doi: 10.1186/s12877-023-04229-x (PMC10463345; doi:10.1186/s12877-023-04229-x)
Supplement: Supplementary file 2 — Additional file 2. Detailed search strategy used to identify relevant studies. [file 12877_2023_4229_MOESM2_ESM.pdf]

Additional file 2 Detailed search strategy used to identify relevant studie

Concept 1:<br>Facilitators	Concept 2:<br>Barriers	Concept 3:<br>Mental health	Concept 4:<br>Help-seeking
promot*<br>OR<br>facilitator*<br>OR<br>support*<br>OR<br>encourag*<br>OR<br>Help*<br>OR<br>Assist*<br>OR<br>Mediat*<br>OR<br>Enabl*<br>OR<br>Advanc*	barrier*<br>OR<br>hurdle<br>OR<br>obstruct*<br>OR<br>Limit*<br>OR<br>Stop<br>OR<br>Obstacle<br>OR<br>Impediment<br>OR<br>Hindrance<br>OR<br>Hinder*<br>OR<br>Drawback*<br>OR<br>Obstruction*<br>OR<br>Deterrent<br>OR<br>Complications<br>OR<br>Difficult*<br>OR<br>Stressor*	“mental health*”<br>OR<br>“mental health disorder*”<br>OR<br>psychiatr*<br>OR<br>“Mental health problems”<br>OR<br>“Mental health experiences”<br>OR<br>“mental illness*”<br>OR<br>“mental health disease*”<br>OR<br>“Mental disturbance”<br>OR<br>“Mental health issues”<br>OR<br>“Mental health complications”<br>OR<br>“Mental sickness”<br>OR<br>Derangement<br>OR<br>“Emotional instability”<br>OR<br>“Mental disease”<br>OR<br>“Emotional disorder”<br>OR<br>“Mental instability”<br>OR	“Help-seek*”<br>OR<br>“seek* help”<br>OR<br>“Help-seeking”<br>OR<br>“Help-seeking behav*”<br>OR<br>“Help-seeking attitude*”<br>OR<br>“Help-seeking intention*”<br>OR<br>“Aid seeking”<br>OR<br>“Assistance seeking”<br>OR<br>“Service seeking”<br>OR<br>“Request assistance”<br>OR<br>“Ask for help”<br>OR<br>“Ask for assistance”<br>OR<br>“Ask for support”<br>OR<br>“Seek support”<br>OR<br>“Look for assistance”<br>OR<br>“Ask for guidance”<br>OR

		“Mental derangement”<br>OR<br>“Psychological disorder”<br>OR<br>“Psychological maladjustment”<br>OR<br>“Mental unbalance”<br>OR<br>“Mental health support”<br>OR<br>“Mental health care needs”<br>OR<br>“Mental deterioration”<br>OR<br>Depression<br>OR<br>Anxiety<br>OR<br>“Generalized anxiety disorder*”<br>OR<br>“Psychological Distress”<br>OR<br>“Emotional distress”<br>OR<br>“Psychological problem*”<br>OR<br>“Psychological disturbance*”<br>OR<br>“Mental Distress”<br>OR<br>“Mental suffering”<br>OR	“Look for guidance”<br>OR<br>“Look for support”<br>OR<br>“Require assistance”<br>OR<br>“Seek guidance”<br>OR<br>“Find help”<br>OR<br>“Seek assistance”<br>OR<br>“Request support”<br>OR<br>“Plea for help”<br>OR<br>“Mental health information seeking”<br>OR<br>“Psychological therap*”<br>OR<br>“Psychological treatment”<br>OR<br>“Depression help-seeking”<br>OR<br>“Anxiety help-seeking”<br>OR<br>“Help-seeking behavi*r”<br>OR<br>“Online help-seeking”<br>OR<br>“Online mental health service*”<br>OR

		"Psychological disabilities"<br>OR<br>"Emotional suffering"	"Accessing services for mental health conditions"<br>OR<br>"Mental health service use"<br>OR<br>"Mental health service*"<br>OR<br>"EMental health service use"<br>OR<br>"Mental health service needs"
