# Supplementary material for: Barriers and facilitators of older adults for professional mental health help-seeking: a systematic review
Source: BMC Geriatr. 2023 Aug 25;23:516. doi: 10.1186/s12877-023-04229-x (PMC10463345; doi:10.1186/s12877-023-04229-x)
Supplement: Supplementary file 3 — Additional file 3. NEWCASTLE - OTTAWA QUALITY ASSESSMENT SCALE. [file 12877_2023_4229_MOESM3_ESM.docx]

**NEWCASTLE - OTTAWA QUALITY ASSESSMENT SCALE**

**(adapted for cross-sectional studies)**

**Selection:**

1. Representativeness of the sample:
   1. Truly representative of the average in the target population. * (all subjects or random sampling)
   2. Somewhat representative of the average in the target group. * (non-random sampling)
   3. Selected group of users/convenience sample. 0
   4. No description of the derivation of the included subjects. 0
2. Sample size:
   1. Justified and satisfactory (including sample size calculation). *
   2. Not justified. 0
   3. No information provided. 0
3. Non-respondents:
   1. Proportion of target sample recruited attains pre-specified target or basic summary of non-respondent characteristics in sampling frame recorded. *
   2. Unsatisfactory recruitment rate, no summary data on non-respondents. 0
   3. No information provided. 0
4. Ascertainment of the exposure (risk factor):
   a) Validated measurement tool. **
   b) Non-validated measurement tool, but the tool is available or described.*
   c) No description of the measurement tool. 0

**Comparability:** (Maximum 2 stars)

The subjects in different outcome groups are comparable, based on the study design
or analysis. Confounding factors are controlled.
a) The study controls for the most important factor (select one). *
b) The study control for any additional factor. *

c) The study does not investigate potential confounders. 0

**Outcome:**

1) Assessment of the outcome: 
a) Independent blind assessment. ** 
b) Record linkage. ** 
c) Self report. * 
d) No description. 0

1. Statistical test:
   1. Statistical test used to analyse the data clearly described, appropriate and measures of association presented including confidence intervals and probability level (p value). *
   2. Statistical test not appropriate, not described or incomplete. 0

Cross-sectional Studies:

Very Good Studies: 9-10 points

Good Studies: 7-8 points

Satisfactory Studies: 5-6 points

Unsatisfactory Studies: 0 to 4 points
