# Supplementary material for: Barriers and facilitators of older adults for professional mental health help-seeking: a systematic review
Source: BMC Geriatr. 2023 Aug 25;23:516. doi: 10.1186/s12877-023-04229-x (PMC10463345; doi:10.1186/s12877-023-04229-x)
Supplement: Supplementary file 4 — Additional file 4. Newcastle-Ottawa Scale of included cross-sectional studies. [file 12877_2023_4229_MOESM4_ESM.pdf]

Additional file 4: Newcastle-Ottawa Scale of included cross-sectional studies

Newcastle-Ottawa Scale of included cross-sectional studies								
**Study**	**Selection**				**Comparability**	**Outcome**		**Total quality score**
	**Representativeness of sample**	**Sample size**	**Non-respondents**	**Ascertainment of exposure**	**Control for confounding factors**	**Assessment of outcome**	**Statistical test**	
Anderson et al., 2017	1	0	0	1	2	1	1	6
Chai et al., 2021	1	1	0	2	2	1	1	8
Brenes et al., 2015	1	1	1	2	2	1	1	9
Blais et al., 2015	1	0	0	2	2	1	1	6
Sorkin et al., 2016	1	1	1	1	2	1	1	8
Holvast et al., 2012	1	1	0	2	2	1	1	9
Sorkin et al., 2011	1	1	1	2	2	1	1	9
Perez-Zepeda et al., 2013	1	0	0	1	2	1	1	6
